# Nasal decolonization in total joint arthroplasty: current state of evidence

**DOI:** 10.5194/jbji-11-299-2026

**Published:** 2026-05-22

**Authors:** Andrea Zampoli, Ghazal Pourbozorg, Goksel Dikmen, Ibrahim Tuncay, Javad Parvizi

**Affiliations:** 1 Operative Research Unit of Orthopaedic and Trauma Surgery, Fondazione Policlinico Universitario Campus Bio-Medico, Via Alvaro del Portillo 200, 00128 Rome, Italy; 2 Research Unit of Orthopaedic and Trauma Surgery, Fondazione Policlinico Universitario Campus Bio-Medico, Rome, Italy; 3 International Consensus Meeting (ICM), Department of Orthopedics and Trauma Surgery, University Medical Center Schleswig-Holstein, Campus Kiel, Arnold-Heller-Straße 3, 24105 Kiel, Germany; 4 International Joint Center, Acibadem University Hospital, Istanbul, Türkiye

## Abstract

Periprosthetic joint infection is a major cause of morbidity and economic cost after total joint arthroplasty, with *Staphylococcus aureus* consistently identified as the most common pathogen causing surgical site infection (SSI) and periprosthetic joint infections (PJIs). Nasal mucosa is one of the principle reservoirs for *S. aureus*. Molecular epidemiology investigations have found concordance between nasal organisms and infecting strains, implying that many postoperative infections arise endogenously. Over the last decade, various clinical trials, institutional protocols, and meta-analyses have evaluated the efficacy of nasal screening and decolonization of *Staph aureus*. Recent evidence suggests that the success of infection prevention procedures is dependent on the reliability and consistency of decolonization rather than screening alone. This narrative review summarizes current evidence on nasal decolonization in total joint arthroplasty, including epidemiological data, methods of screening, and accessible therapy choices to suggest practical and reproducible infection control measures.

## 1 Background

Periprosthetic joint infection (PJI) is a devastating complication following total hip and knee arthroplasty, resulting in considerable morbidity and economic burden (Parvizi et al., 2010). While multiple patient- and procedure-related factors contribute to infection risk in elective orthopaedic surgery, identifying modifiable risk factors remains central to prevention efforts (Kunutsor et al., 2016). *Staphylococcus aureus*, encompassing both methicillin-sensitive (MSSA) and methicillin-resistant (MRSA) strains, is the predominant pathogen implicated in surgical site infections (SSIs) and PJIs after total joint arthroplasty (TJA) (Skråmm et al., 2014). Nasal colonization serves as the primary reservoir for *S. aureus*, and molecular typing studies indicate that most postoperative infections originate from the patient's endogenous nasal flora. Approximately 20 %–30 % of patients scheduled for elective orthopaedic procedures are colonized with *Staph aureus*, which increases the likelihood of postoperative infection (Stambough et al., 2017; Zhu et al., 2020). Importantly, nasal carriage constitutes a modifiable risk factor. Multiple clinical studies (Ribau et al., 2021a; Stambough et al., 2017; Zhu et al., 2020), institutional protocols, and systematic reviews have demonstrated that preoperative *S. aureus* screening and nasal decolonization are associated with reduced risks of SSI and, in some instances, PJI following TJA. Ribau et al. (2021a) demonstrated that, in elective TJA, lack of preoperative nasal decolonization significantly increased the risk of *S. aureus* infection (RR = 2.18 ± 0.41). Despite accumulating evidence supporting these interventions, uncertainties persist regarding optimal patient selection, screening techniques, decolonization regimens, timing and duration of therapy, agent choice, and integration into standard arthroplasty care pathways. This narrative review synthesizes current evidence on nasal decolonization in TJA and outlines a pragmatic, decision-focused approach for clinical implementation.

## 2 Correlation between nasal colonization and infection after TJA

*S. aureus* infection is associated with prolonged hospitalizations, increased mortality, higher healthcare costs, and a higher risk of readmission within 1 year after total hip and knee arthroplasty (De Buys et al., 2023; Weiser and Moucha, 2015). Approximately 4 % of patients undergoing total hip or knee arthroplasty require hospital readmission within 1 year because of infectious complications (Zawadzki et al., 2017). *S. aureus* represents the leading pathogen across the spectrum of postoperative infections after TJA (Weinstein et al., 2023). Analyses derived from large national surveillance systems, including data from the Centres for Disease Control and Prevention (CDC) (National Healthcare Safety Network) (NHSN) (UK Health Security Agency, 2026), indicate that *Staphylococcus aureus* accounts for approximately 60 %–63 % of deep SSIs and PJI following hip and knee replacement. According to the UK's yearly national surveillance report (UK Health Security Agency, 2026), *Staphylococcus aureus* is the most common cause of postoperative infections. MSSA accounts for around 17.2 % of confirmed cases, while MRSA accounts for approximately 2.4 %, demonstrating its continued importance despite geographical disparities in rates. Molecular typing studies consistently reveal a high degree of genetic concordance between nasal carriage and infecting *S. aureus* isolates in orthopaedic surgery. These findings support the hypothesis that most postoperative *Staphylococcus* infections originate endogenously (Ma et al., 2022; Piewngam and Otto, 2024; Troeman et al., 2023).

Nasal colonization is a modifiable patient-related risk factor in elective TJA and can be effectively addressed through standardized preoperative screening and decontamination protocols. Several studies (Bianco Prevot et al., 2024; Lin et al., 2021) have demonstrated that patients undergoing nasal decolonization experience a lower incidence of postoperative SSI and a reduced rate of PJI compared to untreated control groups.

## 3 Screening and decolonization versus universal decolonization for patients undergoing TJA

Preoperative screening for *S. aureus* is best regarded as an implementation-dependent strategy rather than a universal requirement in TJA. Screening for MRSA prior to elective orthopaedic surgery may constitute sound clinical practice when informed by local epidemiological data. The 2024 ESCMID guidelines (Righi et al., 2024) limit this recommendation to centres where screening is feasible and timely, allowing results to be returned early enough to guide decolonization without delaying surgery. Furthermore, the guideline indicates that current evidence does not support screening as an isolated intervention to reduce SSI caused by *S. aureus* but instead recommends its use as part of a combined screen-and-treat protocol. Similarly, the 2025 International Consensus Meeting (ICM) (Yildiz et al., 2025) found no evidence to indicate that universal MRSA screening conferred much clinical benefit in reducing SSI/PJI after major orthopaedic procedures. Consequently, the decision between targeted and universal screening should be tailored to local MRSA prevalence, laboratory turnaround times, and the ability to incorporate results into immediate perioperative management. A screen-and-treat strategy limits decolonization to documented carriers, avoiding unnecessary treatment in non-colonized patients. However, culture-based screening requires adequate turnaround time, while rapid molecular assays may reduce delays but remain limited by cost and workflow constraints. Current evidence indicates that the clinical benefit of preoperative infection prevention strategies in TJA derives primarily from nasal decolonization itself rather than from screening. While the protective effect of decolonization in TJA is well established, the optimal implementation strategy of targeted decolonization based on screening versus universal decolonization remains less clearly defined in the orthopaedic literature. Stambough et al. (2017) compared a screen-and-treat strategy with a universal decolonization approach in patients undergoing TJA, demonstrating a significant reduction in both 90 d overall infections and *S. aureus* infections in the universal decolonization group. Cost analysis indicated net savings exceeding USD 700 000 in the universal decolonization group. Additionally, Kerbel et al. (2018) assessed economic thresholds for cost-effectiveness and found that screen-and-treat treatment strategies require substantially higher absolute risk reductions to be cost-effective compared to universal decolonization protocols. A recent systematic review and meta-analysis by Ribau et al. (2021b) found that universal decolonization is both the most cost-effective strategy and the most effective at reducing PJI. This approach also eliminates the risk of untreated carriers resulting from limitations in screening sensitivity or delays in result availability. Nevertheless, the universal use of topical antibiotics raises concerns about the potential emergence of mupirocin resistance, an issue that most economic models do not fully address (Hetem et al., 2016). To reduce this risk, antiseptic alternatives such as octenidine or intranasal povidone-iodine have been proposed and are increasingly implemented (Hammond et al., 2023; Abolghasemi et al., 2025; Anderson et al., 2015). In alignment with this evidence, the ICM 2025 (Yildiz et al., 2025) recommends routine nasal decolonization for all patients undergoing major orthopaedic procedures, preferably utilizing a non-antibiotic antiseptic agent.

## 4 Options for nasal decolonization

Intranasal mupirocin has been the primary topical antibacterial agent for nasal decolonization, demonstrating activity against *Staphylococci* and *Streptococci* (Ward and Campoli-Richards, 1986). Prior studies have shown that intranasal mupirocin significantly reduces nasal *S. aureus* burden, supporting its inclusion in decolonization protocols (Perl et al., 2002). Standard regimens recommend 2 % mupirocin applied to the anterior nares twice daily for 5 d (Septimus, 2019). Despite high initial efficacy, recolonization or persistence of colonization is common when mupirocin is used. Several studies report elimination of nasal *S. aureus* in 91 % of treated patients within 96 h, but recurrence rates exceed 50 % within 2 to 6 months (Baede et al., 2022; Fernandez et al., 1995; Septimus, 2019). Decolonization failure is closely linked to mupirocin resistance, as resistant strains are more likely to persist despite topical therapy, while susceptible strains are more likely to be eradicated in the short term (Patel et al., 2009; Premanand et al., 2023). Reported resistance rates are approximately 10 % in *S. aureus* and 15 % in MRSA, yet routine resistance testing is infrequent (Fouad et al., 2019; Jones et al., 2007; Rudresh et al., 2015). The CDC (Centers for Disease Control and Prevention, 2026) recommends chlorhexidine gluconate (CHG) wash as an adjunct to mupirocin to reduce bacterial load at extra-nasal sites. Alternative topical agents, such as neomycin or fusidic acid, have been proposed, but comparative clinical evidence remains limited (Smith and Herwaldt, 2023). In TJA, iodine- or chlorhexidine-based agents are recommended to support decolonization and minimize the risk of antibiotic resistance (Fernández-Rodríguez et al., 2024; Righi et al., 2024). Non-antibiotic topical agents have also been investigated as alternative strategies for nasal decolonization. Povidone-iodine (PVP-I) demonstrates broad antimicrobial activity against Gram-positive and Gram-negative bacteria, including MSSA and MRSA (Septimus, 2019). Early in vitro studies identified PI as a potential alternative to mupirocin, although nasal secretions can reduce its antimicrobial efficacy (Hill and Casewell, 2000). In a randomized, placebo-controlled trial, Rezapoor et al. (2017) found that a 5 % PVP-I-based nasal antiseptic achieved significantly greater *S. aureus* decolonization at 4 h compared to off-the-shelf PVP-I ( P = 0.003). Phillips et al. (2014) compared intranasal mupirocin with PVP-I in patients undergoing TJA and spine surgery. Although postoperative culture negativity was higher with mupirocin than with PVP-I (92 % vs. 54 %), the per-protocol analysis showed that *S. aureus* deep SSI occurred in 5 of 763 procedures in the mupirocin group and in 0 of 776 procedures in the PVP-I group ( P = 0.03). Similarly, Ghaddara et al. (2020) showed that a single intranasal application of 10 % PVP-I significantly reduced MRSA colony counts at 1 to 6 h, but the effect was not sustained at 12 to 24 h. Current evidence supports nasal PVP-I as a well-tolerated, non-antibiotic alternative for perioperative SSI prevention, with its primary benefit limited to the immediate preoperative period rather than long-term decolonization (Septimus, 2019; Smith and Herwaldt, 2023). This limitation has prompted interest in broader, resistance-free techniques suitable for general use. Alcohol-based nasal antiseptics offer a non-antibiotic decolonization strategy with rapid-onset, broad antimicrobial activity, and no risk of resistance (Hoffmann et al., 2024). Hoffmann et al. (2024) confirmed bactericidal activity against Gram-positive, Gram-negative, and fungal organisms, supporting a microbiome-modulating approach. Randomized trials (Kanwar et al., 2019; Steed et al., 2014) indicate that a single dose provides only transient suppression, while repeated dosing is necessary to maintain bacterial reduction. Clinical studies have demonstrated that alcohol-based antiseptics, when used perioperatively or postoperatively, is associated with reduced SSI rates, including TJA populations, with high adherence and favourable safety profiles (Bostian et al., 2023; Mullen et al., 2017). A meta-analysis by Hoffmann et al. (2024) found that alcohol-based antiseptic was more effective than mupirocin- and iodophor-based agents in preventing SSIs, with superior outcomes compared to mupirocin (OR ≈ 4.1) and iodophors (OR ≈ 3.0). Retapamulin, a topical pleuromutilin antibiotic active against MSSA and MRSA, has been evaluated as a salvage agent in mupirocin-resistant carriers. A randomized, placebo-controlled trial reported higher short-term decolonization rates, but the effect was not durable, limiting its use to selected cases (Patel et al., 2019).

## 5 Conclusions

Nasal colonization with *S. aureus* is a significant risk factor for postoperative infections following TJA. However, management strategies remain inconsistent. Clinical experience and implementation studies indicate that universal decolonization has a greater impact on preventative effectiveness than screening. Targeted screening may be appropriate in high-volume institutions with access to rapid tests, but logistical problems, delays in delivering test results, and false-negative results are serious disadvantages of screening. Thus, universal decolonization is a more practical and reliable option. Intranasal mupirocin is still effective in reducing nasal bacterial load, but the rise in resistance detracts from the efficacy of this agent. Based on the above information, the following key principles may be considered for patients undergoing arthroplasty:

- Universal nasal decolonization should be established as a standard component of infection prevention protocols for all patients, rather than being applied sporadically or at the discretion of individual clinicians.
- Although mupirocin has been shown to be an effective decolonization agent, due to rising resistance and antimicrobial stewardship, consideration should be given to non-antibiotic antiseptic agents for universal decolonization.

## Data Availability

No original research data were generated or analyzed in this study. All data and evidence discussed in this narrative review are derived from previously published articles cited in the reference list. Therefore, no additional dataset is available for deposition in a public repository.
